# The Association between Physical Activity, Self-Compassion, and Mental Well-Being after COVID-19: In the Exercise and Self-Esteem Model Revised with Self-Compassion (EXSEM-SC) Perspective

**DOI:** 10.3390/healthcare11020233

**Published:** 2023-01-12

**Authors:** Ming-Yu Claudia Wong, Hong-Wang Fung, Guangzhe Frank Yuan

**Affiliations:** 1 Department of Health and Physical Education, The Education University of Hong Kong, Hong Kong, China; 2 Department of Social Work, Hong Kong Baptist University, Hong Kong, China; 3 South Carolina Smart State Center for Healthcare Quality, Arnold School of Public Health, University of South Carolina, Columbia, SC 29208, USA; 4 Department of Health Promotion, Education, and Behavior, Arnold School of Public Health, University of South Carolina, Columbia, SC 29208, USA

**Keywords:** self-compassion, physical activity, mental well-being, adolescents

## Abstract

During the great life-altering challenges brought by Coronavirus 2019, school closures and lack of access to exercise and social interactions may have increased students’ negative emotions. The current research acts as a follow-up study to the development of the EXSEM-SC, using the Repeated Measures Panel Analysis Framework (RMPAF) to examine the stability of the model in revealing the relationship between physical activity, self-compassion, and mental well-being among Hong Kong adolescents. It is also aimed at examining the changes in physical activity, self-compassion, and mental well-being among Hong Kong adolescents between, before, and after the peak of COVID-19 using the EXSEM-SC Model. The RMPAF has involved 572 (60% Female, *Mage* = 13.63, SD =1.31) Hong Kong secondary school students. Furthermore, using the abductive qualitative approach, a total of 25 (*Mage* = 14.84, *SD* = 1.40) students were involved in the in-depth interviews to further investigate the relationships within the EXSEM-SC. The quantitative results showed that the relationship between physical activity and self-compassion could be demonstrated by the EXSEM-SC, with a satisfactory goodness-of-fit index in the SEMs, as well as satisfying model construct consistency. Moreover, it showed no significant differences in the level of physical activity, self-compassion, and mental well-being during and after the peak of COVID-19. The qualitative results demonstrated two new categories within the EXSEM-SC variables, which are personality traits and injuries experiences. With the stability of the EXSEM-SC model among adolescents, it is expected that the physical activity intervention, which is based on the EXSEM-SC model, could also aim at easing Hong Kong adolescent’s mental health issues. In addition, in terms of generating a long-term impact among students, the physical activity and self-compassionate intervention should be promoted among schools. However, the quantitative properties of the two new categories in the qualitative outcomes should be involved in future investigation.

## 1. Introduction

With its ongoing outbreak, the Coronavirus disease 2019 pandemic has not only caused a higher risk of death from the viral infection, but also led to great life-altering challenges. As the outbreak continues, youth’s psychological problems may increase during the COVID-19 pandemic [1]. Youth are likely to be experiencing worry, anxiety, and fear [2]. School closures, lack of access to exercise and social interactions, boredom, or family poverty may increase students’ negative emotions because they have less social support, which is essential for good mental health and well-being [3]. A cross-sectional study was conducted to assess a two-time point comparison between the months preceding the COVID-19 outbreak and two months after the social distancing and online schooling measures were implemented [4]. It indicated that adolescents showed a significant increase in depressive symptoms, anxiety, and poor parent-child relationship after the COVID-19 measures, while the COVID-19-related worries led to a significant decrease in life satisfaction as well [4]. Given that independence from parents and peer interaction are considered as the most important factors towards adolescents’ development [5], school closures and the lack of peer support and interactions are likely to be the pertinent sources of stressors among adolescents, which affect their self-concept shaping and the estimation of self-worth [6], thus leading to high risk of vulnerability. Furthermore, adolescents have often been labelled as emotional and sensitive that their self-regulatory system is considered not yet mature until early adulthood [7]; therefore, fostering self-compassion among adolescents is considered a protective factor against adolescents’ negative mental impact during COVID-19.

### 1.1. Self-Compassion in COVID-19

Self-compassion has been shown to improve adolescents’ sense of well-being and mental health through providing oneself with self-kindness and reducing negative self-esteem [8,9]. Self-compassion is also seen as a coping mechanism in transforming negative feelings into neutral emotions, and towards a more productive emotion for oneself. It was discovered that a high level of self-compassion was associated with more emotion-focused coping mechanisms utilization, such as a positive reinterpretation and accepting their shortcomings [10]. Hence, self-compassion can act as a protective role in positive interventions to enhance the coping strategies of adolescents in regards to reacting to adversity during COVID-19 [11]. COVID-19-related research studies indicated that people who resulted in low levels of self-compassion tended to show anxiety and depression [12], and people with high levels of self-compassion tended to show better life satisfaction, despite self-quarantine [13]. Furthermore, a Hong Kong research study found that self-compassion is critical in influencing a person’s adjustment to unprecedented problems [14], with self-compassion demonstrating a strong moderating influence between threats and perceived benefits of the pandemic (i.e., more time for rest and relaxation, learning new skills or knowledge), whereas persons with a higher degree of self-compassion exhibited a less positive relationship with the pandemic.

### 1.2. Physical Activity and COVID-19

During COVID-19, studies revealed the negative effects of social distancing measures, quarantining measures, and the lack of social support on adolescents’ mental health. Several studies found a connection between social distancing measures, depression, and anxiety [15]. Furthermore, research has shown that reduced physical activity and social activities as a result of COVID-19 have led to higher scores of anxiety and depressive symptoms as well [16]. A study found that social restrictions had led to a drop in teenagers’ physical activity participation, prolonged sedentary behavior, owing to online schooling and screen time, and irregular sleep patterns, creating long-term psychological health risks among adolescents during COVID-19 [17]. Among the Asian population studies, they revealed similar results on the reduced physical activity and increased sedentary behaviour among Chinese youths during COVID-19 [18]. As such, they revealed the importance of physical activity and a healthy lifestyle in mental health during COVID-19 [19].

### 1.3. The Relationship between Physical Activity and Self-Compassion

Given that the essential function of self-compassion in enhancing mental well-being has been validated, and programs such as the American College of Sports Medicine’s Exercise is Medicine^®^ represent the greatest way of preventing mental health problems [20], the impacts of regular physical activity on lowering mental health risk and boosting positive well-being cannot be refuted [21,22,23,24] conducted a comprehensive review and meta-analysis of the link between self-compassion and physical activity, and they found substantial relationships between self-compassion, physical activity, and physical activity intention. Moreover, Wong [24] have developed the model, named the Exercise Self-Esteem Model Revised with Self-Compassion (EXSEM-SC [24]), which significantly indicated the significant conceptualization among physical activity and self-compassion, with the involvement of exercise self-efficacy and body compassion. The EXSEM-SC Model has kept the variables of exercise self-efficacy, while replacing physical competence and acceptance with body compassion. It was anticipated that body compassion would be a more distinctive method to demonstrate the level of self-compassion of the physical self [25]. The significant result of the EXSEM-SC model demonstrates the possible influences of physical activity participation on adolescents’ level of self-compassion. The EXSEM-SC version was changed based on Sonstroem and Morgan Exercise and Self-Esteem Model (EXSEM)developed in 1989, which at the start indicated the impact of bodily activity or exercise participation on character self-concept [26]. Despite the lack of evidence demonstrating the umbrella construct of self-compassion above self-esteem, Neff’s argument on the mental hazards induced by self-esteem (i.e., self-enhancement bias, narcissism, and social comparison) and Deci and Ryan’s conjecturing self-compassion as a type of “True self-esteem” [10,27,28,29] encourage the examination of self. As a result, the construction and adaptation of the EXSEM-SC model in showing the association between physical exercise and self-compassion in the current study is regarded robust and illustrative.

### 1.4. Research Gap and Purpose

Participating in self-based physical exercise is seen as autonomously accomplished under the notion of self, which could greatly lower emotional vulnerability during COVID-19. However, it is worth mentioning that only a few studies with mixed results compared the results of two-time points to investigate the impact of the COVID-19 pandemic on mental health and self-compassion in adolescents (during the peak of COVID-19 versus post-COVID-19 social distancing measures). Only two studies identified a significant negative correlation between COVID-19-related concerns, such as spending less time with friends and schooling, and negative mental well-being, such as loneliness, depression, and anxiety, among these few repeated measures or longitudinal studies [30,31]. The remaining studies, the majority of which looked at the Chinese population, found no significant differences in mental health status, such as depression, anxiety, stress, or suicidal thoughts, between the two time points [32,33,34].

Given that the COVID-19 pandemic has affected both adolescents’ physical activity and psychological status, while documenting the importance of self-compassion in coping with adversity during COVID-19, the current research is aimed to examine the changes in physical activity, self-compassion, and mental well-being among Hong Kong adolescents between the peak of COVID-19 and after a certain release of the social distancing measures, using the Exercise Self-Esteem Model Revised with Self-Compassion (EXSEM-SC [24]). In addition to reviewing the impact of COVID-19, this research is also seen as a follow-up study of the development of EXSEM-SC, which is to investigate the stability and reliability of the model in demonstrating the relationship between physical activity, self-compassion, and mental well-being among Hong Kong adolescents. Furthermore, as a follow-up study of the EXSEM-SC, this study has conducted a qualitative interview to exploratively analyse the EXSEM-SC, as to explore the underlying interaction between physical activity and self-compassion. The qualitative study has abductively explored the EXSEM construct with self-compassion, which enabled the deductive analysis of the EXSEM-SC model, as well as the inductive analysis of the possible newly derived variables underneath the relationship between physical activity and self-compassion.

Based on the purpose of the study, the hypotheses of the study are,

**Hypothesis** **1.** *There are significant mean differences between Time 1 and Time 2 (during COVID) in terms of the level of physical activity and self-compassion.*

**Hypothesis** **2.** *A good-fit model is found for the EXSEM-SC when using a Repeated Measures Panel Analysis Framework.*

**Hypothesis** **3.** *The interview outcomes are found to deductively support the EXSEM-SC and inductively derive additional variables for future modification.*

## 2. Methods

### 2.1. Participants and Procedures 

Around 1000 students were recruited through convenience sampling in response to the sample size rule of thumb of structural equation modelling in the previous study [24]. Three government subsidized schools from the New Territories (n = 579), one school from Kowloon (n = 231), and one school from Hong Kong Island (n = 285) were recruited, as described in the earlier study by Wong et al. (2021). Notably, regardless of their degree of physical activity, all eligible secondary school students were able to participate in the current study. Students with any type of disability (including motor, visual, and hearing impairments, as well as intellectual and social difficulties) or who had been diagnosed with a mental or cognitive disorder were excluded from the study. The survey [24] was first conducted during the third wave of COVID-19 in Hong Kong (mid-to-late October 2020). In order to perform the repeated measure panel analysis, the students were invited to participate in the research survey after a 4 to 5 month interval for the retest, in which half-day face-to-face schooling started to resume. However, among the 1097 students involved in the previous model testing [24], only 589 students participated in the re-test survey. Among the 589 students, 20–30 participants were invited to participate in the in-depth interview as well. The students invited to be in the interview were required to be (1) Secondary school students, regardless of their physical activity participation level, and (2) students who were able to communicate fluently in either English or Chinese. Exclusion criteria were as follows: (1) Students with any kind of disability (i.e., motor impairment, visual or hearing impairment, intellectual or social disability) or diagnosed with any mental or cognitive illness, and (2) students who had undergone surgery that prevented them from participating in physical activity. In addition to this, students with past or current mental health issues, such as anxiety, stressfulness, or non-diagnosed depression, needed to seek school teachers’ and parents’ approval and had to be accompanied in order to participate in the interviews, or else they were excluded from the study.

### 2.2. Transparency and Openness 

The University Research Ethics Committee granted ethical permission prior to the start of the study. After permission was granted, all data were collected at the schools. Before taking the questionnaire, all participants were required to sign a permission form stating that they understood the study’s confidentiality agreement and that they were free to quit at any moment. Prior to data collection, all parents’ informed consents were obtained through schools. We report how we determined our sample size, all data exclusions (if any), all manipulations, and all measures in the study, and we follow JARS [35]. All data, analysis code, and research materials are available upon request. 

## 3. Measures

### 3.1. Self-Compassion

To determine the level of self-compassion, the Chinese version of the Self-compassion Scale [10,36] was used. The self-compassion measure is a 26-item, 5-point Likert scale derived from the 6 self-compassion components: self-kindness, common humanity, mindfulness, self-judgment, isolation, and over-identification (1 = nearly never, 5 = almost always). The test-retest reliability of the Chinese version of the self-compassion questionnaire was 0.89, with a Cronbach’s alpha of over 0.80 among Chinese students [36,37].

### 3.2. Body Compassion

The Chinese-translated body compassion scale, which is able to enlighten both physical perception and self-compassion, was adopted to measure the level of body compassion. The body compassion scale is a 23-item scale with 3 subscales: defusion, common humanity, and acceptance. The items were rated on a 5-point scale (1 = almost never, 5 = almost always [10,38]. The Chinese-translated body compassion scale [24] showed satisfactory internal consistency and test-retest reliability, as well as adequate goodness-of-fit results, with X² (465.64)/227 = 2.05, *p* < 0.001, CFI = 0.916, TLI = 0.906, SRMR = 0.071, RMSEA = 0.069 (90% CI = 0.06 to 0.078).

### 3.3. Exercise Self-Efficacy

The Exercise Self-efficacy Scale [39,40,41] was used to assess how assured (or confident) individuals were in their ability to maintain a regular exercise regimen under a variety of settings. The Chinese version is based on Bandura’s 17-item, 7-point Likert-type scale [42]. Cronbach’s alpha score for the Chinese version of the scale was 0.944, indicating satisfactory reliability [36].

### 3.4. Physical Activity Subjective Measure 

To acquire self-reported physical activity levels, the Chinese version of the Physical Activity Questionnaire for Adolescents (PAQ-A) was used. The questionnaire has nine questions on a 5-point Likert scale, 8 of which were utilized to calculate physical activity levels. The Chinese version of PAQ-A has a satisfactory Cronbach’s alpha, ranging from 0.82 to 0.85, and a test-retest reliability of 0.81. It also had a moderate correlation value with the accelerometer-based physical activity objective measure [43].

### 3.5. Mental Well-Being

To assess people’s overall mental health, the Chinese version of the Warwick-Edinburgh Mental Well-being Scale Short Form [44] was used. The WEMWBS is a 7-item scale with a 5-point Likert scale for the short version. Despite the fact that it is a short-form scale, the psychological components, such as affective-emotional, cognitive-evaluative, and psychological function, can be demonstrated and studied in a simple manner [45]. With a Cronbach’s alpha of 0.93, an excellent test-retest reliability of 0.84 [44], and good convergent validity (CFI 0.986 and TLI = 0.979), the Chinese version demonstrated satisfactory internal reliability [46].

### 3.6. Interview Setting

The in-depth interviews each lasted for 30–45 min and were conducted via video conferencing (i.e., via Zoom) due to restrictions imposed by the COVID-19 pandemic. To ensure the theoretical sensitivity and trustworthiness of the qualitative research, a triangulation strategy was adopted, with each section of the interview involving more than one interviewer to provide multiple perspectives and follow-up questions. The interviewers (the authors) have no personal relationship with the interviewees, who work outside secondary schools. Yet, the author has been working closely with secondary schools and is familiar with the physical education system in Hong Kong, hence, shows a certain extent of understanding towards the interviewees’ responses and the reality that students are facing.

The interview guide was formulated according to the hypothesized framework of the Exercise and Self-Esteem Model revised with self-compassion. The interview guide involved questions designed to explore the relationship between physical activity and self-compassion from a first-person perspective, through participants’ daily experiences, exercise habits, and their feelings towards physical activities in light of the variables of the Exercise and Self-Esteem Model. This enabled the researchers to investigate how the participants indicated and described the components, as well as revealing the associations between physical activity and self-compassion, and to identify possible variables that were not included in the hypothesized model. 

Moreover, the interview guide contained professional contributions to ensure accuracy and representativeness in illustrating the characteristics of self-compassion for the prospective relationship between physical activity and self-compassion (i.e., content validity). Hence, an expert in self-related concepts was invited to comment on the draft of the interview guide, until consensus was reached between the expert and the author regarding any discrepancies. Furthermore, five secondary school students were invited to participate in a pilot interview to evaluate the choice of words and the clarity of the questions.

## 4. Methodology and Data Analysis 

### 4.1. Repeated Measure Panel Analysis Framework in Structural Equation Modeling 

Through Rstudio [47], the models were constructed and tested in a Structural Equation Modelling Approach, with the full-information maximum likelihood estimator (FIML). The consistency and stability of the variables of the EXSEM-SC model was tested using the Repeated Measure Panel Analysis Framework. The EXSEM model revised with self-compassion is considered as a newly exploring model that variables relationship’s stability is an essential concern. Hence, all the components within the proposed EXSEM structure, including self-compassion, were involved in the repeated measures, in which it is able to analyse whether the model structure is consistent over time and during the COVID-19 pandemic. Subsequently, the consistency of the model was tested based on the repeated measures panel analysis framework. The panel analysis framework allows an examination of both cross-sectional and 2-time point relations simultaneously. In the panel analysis framework, bivariate correlations among all variables were generated, and the first wave of the path analysis model had shown the path coefficients, either direct or indirect effects, between the model components. The second wave of the path analysis model had related to the relationships among the model components across the 2-time point in order to demonstrate the changes in the variables over time. These were identified by analysing the change of the path coefficients, the variation percentage (R²) across a 2-time point, as well as the stability coefficients, for all the components within the panel model. It is acknowledgeable that with fewer variations and a high stability coefficient (0.70 or above), we can consider the structural model as consistent [48]. 

Various additional goodness-of-fit indicators were involved [49] and, with the following level of index, assumed to be a good fit: the minimum fit function (X^2^), having chi-square to the degree of freedom ratio value range 2–5 as an acceptable fit [50]; Comparative Fit Index (CFI; [51]) and Tucker–Lewis index (TLI) rated as 0.90 or above [50]; Standardized Root Mean Square Residual (SRMR) value of 0.08 or below [51]; Root Mean Square Error of Approximation (RMSEA) value as between 0.05–0.08, with a 90% confidence interval [52].

### 4.2. Qualitative Procedures, Methodology and Analysis 

The abductive grounded theory approach of Peirce [53] was utilized as a methodology to explore and investigate the hypothesized EXSEM revised with self-compassion model in order to conceptualize the theoretical construct systematically. The main purpose of abductive reasoning is to uncover the logic by having new ideas come into existence [53]. Tavory and Timmermans [54] summarized that using abductive analysis for generating theory should involve the capture of the narrative view of the targets and unfurl meaning making through interpreting their thoughts and experiences. This is a way leading to the process of theory construction; furthermore, it is an open and continuous process without a standardized meaning of interpretation. Nevertheless, during the abduction, the researcher should first recognize new ideas or breakdown anomaly to create a hypothetical inference, which can provide a meaningful explanation for the latent relations of the selected phenomenon. This is because abduction reasoning is proposed to observe the unknown cause and effect of familiar structures or phenomena [54]. Furthermore, Perice (1931–1958) conceptualized the reason for the abduction process being both logical and a flash of insight [53]. In order to be abductive, the research process should be as follows: First, with recognized phenomenon focus derived from literature reviews, the researcher should imply an established interpretive theory into the data collection process. While during the data synthesis and analysis, the interpretation of the aimed phenomenon should take the established interpretive theory into consideration. This process involves the unpacking and reforming of the dominating theory. Finally, it would then articulate a new interpretative theory from a new angle and a new connection that explains the targeted phenomenon. Abduction helps formulate groundwork interpretation with a theoretical contribution and would extend the bearing of the empirical findings and fill in the limitations of the examined theory. As a result, the abductive grounded theory approach was applied to deductively investigate the EXSEM-SC model and inductively confirm the model, as well as enumerate new variables for possible model modification.

The audio recordings of the interviews were transcribed verbatim, then translated from Chinese to English. The translated transcripts were screened by a second person to eliminate the translation variations, and the verbatim transcripts were returned to participants to verify the accuracy of the interview content, thus enhancing the credibility of the qualitative research. Nvivo 12 (QSR, [55]) was used to manage the empirical data through memo writing, coding, and categorizing until the data saturation was achieved. Conformability of the research was achieved by involving a second coder (that is, someone other than the author) in the coding and synthesis process, and collaboration occurred when the interpretation of data varied. The thematic synthesis approach was used in combination with grounded theory. In applying thematic synthesis, open codes were labelled with “descriptive” themes and then further “analytical” themes for the empirical data generated [56]. In addition, memos were written throughout the synthesis and analytic process to identify, develop, and keep track of theoretical ideas [57]. Furthermore, according to Haig [57], data collection and data analysis are to be done interactively in order to refine and enrich the empirical data subsequently [58,59]. Hence, data analysis has been engaged during the process of data gathering, for further data collection then ensuing data analysis continuously. Moreover, to consolidate and standardize the reporting of our qualitative research, the consolidated criteria for reporting qualitative research (COREQ checklist for interviews) was followed [60].

## 5. Results

Among the 1097 students, only 589 students participated in the re-test survey due to the change of school teaching schedule. While 11 cases with more than 20% missing data were deleted, 6 univariate and multivariate outlier cases were identified and deleted. The remaining missing data were replaced with the mean values of the items. The variables in the data set were normally distributed, with no items greater than 3.3 or less than −3.3. Finally, a total of 572 cases were included in the panel analysis, of which 59.5% and 40% were girls and boys, respectively, and the mean age was 13.63 (SD = 1.31). Additionally, the in-depth interviews were conducted with 25 (15 male, 10 female) secondary school students from both junior forms (n = 11) and senior forms (n = 14), with a mean age of 14.84 (SD = 1.40) years. Demographic information of the participants is indicated in Table 1.

### 5.1. Repeated Measure Panel Analysis Framework

The purpose of building the panel analysis framework is to show the stability and reliability of the Exercise and Self-esteem Model Revised with Self-Compassion and Mental Well-being among the adolescent population. On top of the first (Time 1) path analysis done on EXSEM-SC and EXSEM-SC with mental well-being, participants were involved in a re-test survey (Time 2) four to five months after the first data collection, thus enabling the development of the repeated measure panel analysis framework. Responding to hypothesis 1, Table 2 showed no significant differences in the level of physical activity, self-compassion, and mental well-being between, during and after COVID-19, while only the level of exercise self-efficacy and body compassion showed significant differences among the secondary school students. Table 3 showed the correlation matrix of all the model variables, including both Time 1 and Time 2 variables data. Variables showed a significant correlation with each other at the same time point. Each variable also showed a significant correlation with their respective repeated measure outcome. 

**Figure 1:**
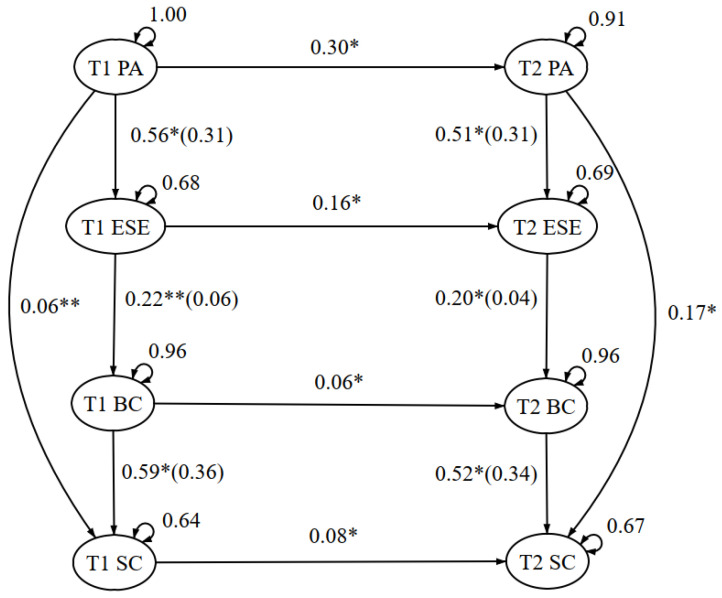
Exercise and Self-Esteem Model Revised with Self-Compassion—Panel Analysis Framework. Note. Standardized coefficients, total variance explained on the outcome (*R*^2^) and errors are presented. *R*^2^ is presented in brackets, in the form of *β*(*R*^2^). * *p* < 0.05, ** *p* < 0.01. T1 PA = Time 1 physical activity; T2 PA = Time 2 physical activity; T1 ESE = Time 1 exercise self-efficacy; T2 ESE = Time 2 exercise self-efficacy; T1 BC = Time 1 body compassion; T2 BC = Time 2 body compassion; T1 SC = Time 1 self-compassion; T2 SC = Time 2 self-compassion.

**Figure 2:**
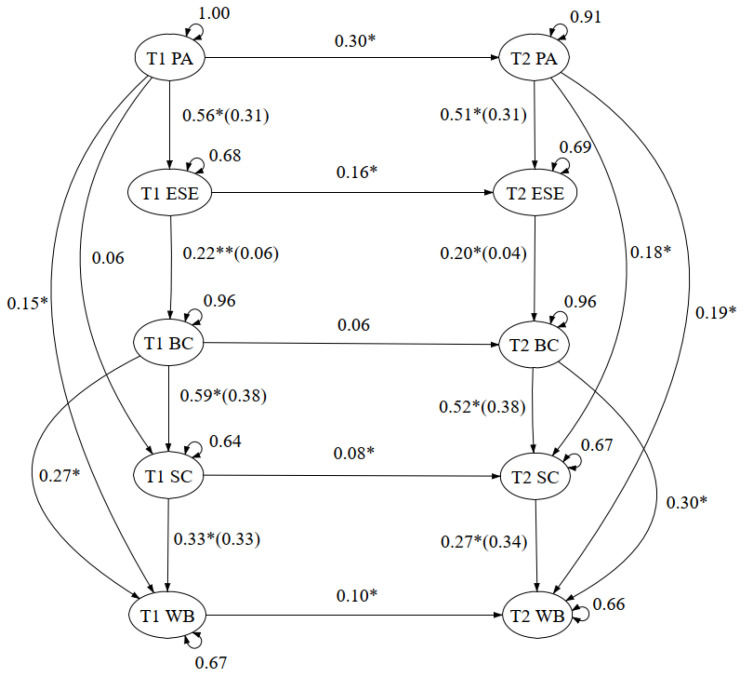
Exercise and Self-esteem Model Revised With Self-Compassion Including Mental Well-being—Panel Analysis Framework. Note. Standardized coefficients, total variance explained on the outcome (*R*^2^) and errors are presented. *R*^2^ is presented in brackets, in the form of *β*(*R*^2^). * *p* < 0.05, ** *p* < 0.01. T1 PA = Time 1 physical activity; T2 PA = Time 2 physical activity; T1 ESE = Time 1 exercise self-efficacy; T2 ESE = Time 2 exercise self-efficacy; T1 BC = Time 1 body compassion; T2 BC = Time 2 body compassion; T1 SC = Time 1 self-compassion; T2 SC = Time 2 self-compassion; T1 WB = Time 1 mental well-being; T2 WB = Time 2 mental well-being.

When comprising and comparing the Time 1 and Time 2 model, the panel analysis framework showed a good fit model in both the EXSEM model revised with self-compassion (X^2^ (60)/16 = 53.75, CFI = 0.957, TLI = 0.957, SRMR = 0.043, and RMSEA = 0.072 (90% CI = 0.053–0.092), as well as the EXSEM model revised with self-compassion and mental well-being (X^2^ (90.84)/26 = 3.49, CFI = 0.956, TLI = 0.94, SRMR = 0.049, and RMSEA = 0.068 (90% CI = 0.053–0.083). Furthermore, the consistency of the path coefficient and the R-square were looked into as well. Referring to Figure 1 and Figure 2, all paths were significant with consistent path coefficients, with all changes within 0.1. Additionally, the R-square changes were within 0.03, which a low R-square change with variation of less than 30% indicated the structural model as consistent [48]. The research outcomes supported hypothesis 2, that the EXSEM-SC model was consistent over time, as well as during COVID-19, when the mental status of participants tended to fluctuate, which also supported the generalizability of the model within different social circumstances. 

### 5.2. Qualitative Interview Outcomes

The 25 participants participated in different kinds of exercise and were at different levels of participation, and some of the participants participated in more than one kind of exercise. Participants’ level of participation was divided into three categories: Leisure-based physical activity, in which physical activity is engaged in for leisure purposes; school-based PA, in which participants had joined the school team and had participated in inter-school competitions; and professional athletes who trained with the Hong Kong team. Participants reported different psychological states and influences during participation in PA. They revealed positive psychological states and benefits during and after participating in PA. In general, participants stated that participation in PA relaxed them, increased their perseverance, and matured them in overcoming difficulties. Unlike such generalities, the Exercise and Self-Esteem model revised with self-compassion revealed particular categories.

## 6. Exercise Self-Efficacy

**Figure 3:**
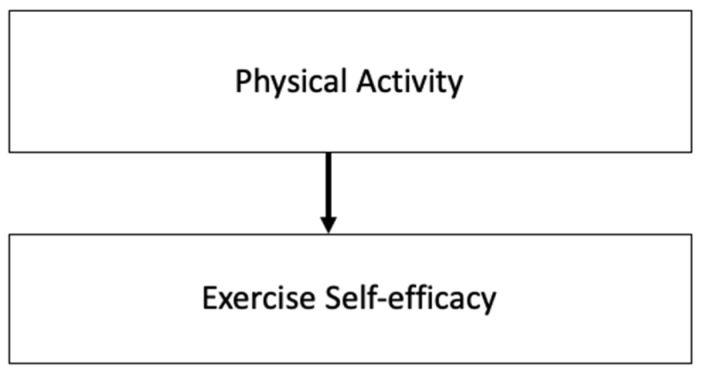
Relationship between Physical Activity and Exercise Self-Efficacy. Note. The figure demonstrate the path from physical activity to exercise self-efficacy, which in line with the original EXSEM.

Exercise self-efficacy refers to the degree to which one can successfully perform, adopt, and maintain an exercise behaviour. Several participants provided relevant statements regarding exercise self-efficacy. Most of the leisure-based PA and school-based PA participants showed positive exercise self-efficacy after experiencing a breakthrough in their exercises (Figure 3).

A school-based PA participant (*CSK1*) stated, “After I have learned a new difficult table tennis technique, I feel accomplished, and have more self-confidence”.

A leisure-time PA participant (*CSK4*) similarly stated, “After I have engaged in regular physical activity, I feel stronger and very happy just like someone who has overcome something difficult”.

In addition, participants showed that the self-efficacy and self-confidence gained through participating in physical activity did not only apply to exercise performance or exercise behaviour maintenance, but also other experiences in their daily lives. 

However, negative exercise self-efficacy was shown in the interview outcomes as well. 

A professional athlete (*SJ1*) stated, “I tend to give up my main event if I fail to perform my personal best in the competition because I will never feel confident in performing it well again in the next competition”.

Negative exercise self-efficacy was not restricted to the high-level competition of professional athletes. A school-based PA participant (*WKF4*) also stated that she would think of herself as a mess if she was not in good condition during exercising. Exercise self-efficacy was seen as a more shaped concept among secondary school students; hence, both young and older adolescents (junior form and senior form students) were able to describe and reveal their thoughts towards exercise self-efficacy, as well as not showing any substantial differences in the effect of physical activity on exercise self-efficacy.

## 7. Self-Compassion—Mindfulness vs. Over-Identified

Under the concept of self-compassion, mindfulness refers to focusing on the present moment and being aware of one’s deficiencies in a neutral emotional state. Participants showed better self-awareness regarding their own difficulties through the calmness and the sense of focus brought on by doing physical activity and the gaining of positive experiences after having engaged in physical activity.

A leisure-based PA participant (*LDF1*) reflected, “Doing exercise helps me focus more on myself, as well as be more attentive when doing other work, so I think that exercise makes me more contemplative”. Therefore, engaging in physical activity led to tranquil contemplation and having a calm mind, which helped participants to be more focused on themselves, as well as more self-aware in their daily issues. Moreover, participants with demonstrably positive exercise self-efficacy, regardless of the level of PA participation, showed the ability to be aware of their deficiencies in a neutral, reflectively way. Hence, they were regarded as being mindful of their daily issues under the concept of self-compassion. However, two of the professional athletes tended to show over-identification towards their deficiencies of performing poorly in their professional sports, which led to negative self-reflection and self-judgment. 

Other than the differences shown among different levels of PA participation, junior form students showed a difference in the use of words, compared to that of senior form students when illustrating the effect of physical activity on being mindful. Junior form students tended to describe their mindful situation as, “I’m not thinking of anything”. or “My brain was plain when I’m doing exercise”, while senior form students tended to use the word “focus”, “being more aware of”, “to reflect”. This indicated that senior form students were more aware than junior form students. However, it is worth noting that, the junior form students were able to be more aware or describe more in details about their inner status after the interviewer provided these keywords in the conversation. 

## 8. Self-Compassion—Self-Kindness vs. Self-Judgment

Self-kindness means that individuals treat themselves with love and kindness, as opposed to self-judgment, which causes individuals to impose cruel judgment on themselves when facing their own deficiencies. 

The current study found that people tended to undergo a process of self-reflection before they engaged in any acts of self-kindness or self-judgment. It was found that, after the mindful process during and after physical activity, participants tended to reflect on their failures, negativities, and deficiencies. 

Some of the leisure-based PA (*WGF6*, *CSK2*) and school-based PA participants (*CSK6*, *LDF4*) stated that they looked at recordings of their workout or competition to figure out and reflect on where to improve. 

After the self-reflection process, participants who were shown to be more positive and optimistic tended to engage in self-kindness. This involved, firstly, performing self-love by treating themselves, such as allowing time for video gaming, reading books, or listening to music, while some participants also mentioned that doing PA was in itself a kind of self-love act. Secondly, it involved showing self-acceptance towards their deficiencies. For instance, a leisure-based PA participant said, “After the process of being aware of what’s wrong with me as well as the process of self-reflection, I tend to accept the current situation, and treat this case as an experience, and find further ways of improvement” (*WGF1*). Thirdly, upon self-acceptance, participants tended to shift the failure as a kind of motivation for improvement, stating, for example, “I tend to be motivated to deal with my other issues after doing exercises” (*WGF1*) and “I will apply this motivated attitude gained from doing exercise to overcome other daily difficulties, telling myself I could pass this difficulty just as I passed the hardship in training” (*LDF4*). Finally, participants with self-kindness would engage in positive self-talk stating, for example, “Everything has a second chance, I can do it over and over again until I do it well” (*WGF6*). As illustrated by the above statements, participants who showed themselves kindness tended not to impose cruel judgments or to self-criticism, but were positive instead. It is also worth noting that participants who showed self-kindness had a positive attitude towards themselves, stating, for example, “As we do our best, that’s enough” (*CSK3*), and “I think the word criticize is too negative for me, therefore, I won’t” (*CSK6*).

However, participants who were perfectionistic and demanding tended to show self-judgment towards their deficiencies before finding ways to improve or to tackle the issue. Participants showed disappointment in themselves by stating, for example, “I would keep looping my negativities, and I don’t know why I still can’t get out of this despite keeping on trying to improve and correct it” (*SJ1*), and “I would wonder whether I’m not working hard enough” (*CSK1*). Moreover, participants who engaged in self-criticism after the awareness process and after reflecting on their deficiencies displayed a cyclical loop of their negativities and liked sinking into a metaphorical hole that was full of their own failures, in which they thought they could not improve. Yet, the level of self-judgement was determined by the importance and the influencing effect of the failure.

A school-based PA participant (*CSK4*) reflected, “I will keep blaming myself and will force myself to do better and get back into good condition”.

A professional athlete (*SJ1*) said, “I have to keep my mind on my training performance, and I will keep focusing on my deficiencies until I find ways to solve them or figure out what’s wrong to me”.

Despite being self-critical, the study showed that participants would go through a transition process between self-criticism and the release of criticism, which entailed engaging in common humanity, thus achieving a positive psychological state.

## 9. Self-Compassion—Common Humanity and Isolation

In the context of self-compassion, common humanity refers to the individual’s ability to not take their life difficulties and failures too personally, but be able to relate to others who are in a similar situation. Most of the participants, both junior and senior form students, showed common humanity when suffering from failings, and they were willing to share their failing with their peers, parents, and teachers when necessary. A few participants noted that as most of their peers might have experienced a similar situation, talking with peers made them feel that their sadness was being shared (*CSK4*, *CSK1*, *LDF5*, *LDF6*). Other participants stated that communicating with teachers or parents could further assist positive self-awareness regarding their deficiencies, thus releasing stress and breaking their attachment to negative emotions (*LDF6*, *LDF7*, *WGF3*, *WGF8*, *SJ2*). However, a minority of participants chose to isolate themselves from their failures, especially after engaging in self-criticism. 

A professional athlete (*SJ1*) stated, “I will tend to escape from that particular event at which I failed to perform at my personal best and try not to compete in that event anymore”.

A participant who engaged in both leisure-based and school-based PA (*WGF4*) also mentioned that she might choose not to face that particular issue until she could find a proper solution.

## 10. The Relationship between Physical Activity and Self-Compassion

**Figure 4:**
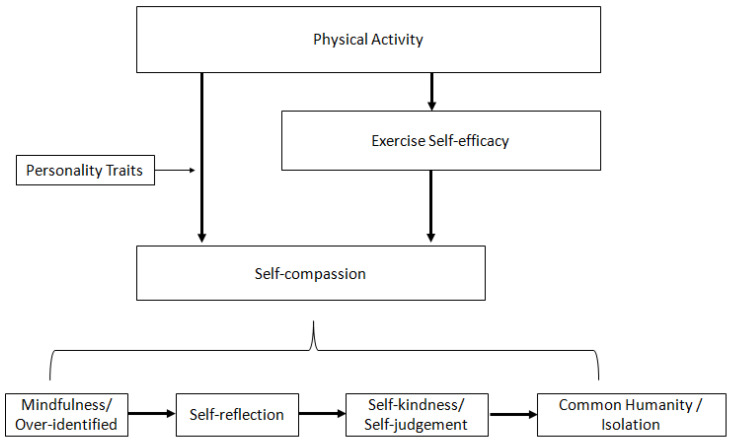
The Relationship between Physical Activity and Self-Compassion. Note. This figure indicated the path from physical activity to self-compassion through exercise self-efficacy. On the other hand, it also indicates the possible mediating effect of personality on physical activity and self-compassion. Additionally, the four sub-categories under self-compassion were shown and demonstrated their processing stage from mindfulness towards common humanity.

Figure 4 shows the conceptual framework of the relationship between physical activity and self-compassion. Firstly, the study outcomes have successfully indicated that physical activity can affect participants’ exercise self-efficacy and lead to self-compassion through engaging in PA, as well as the efficacy gained through PA. In addition, the study has also shown that physical activity can lead directly to individual self-compassion, specifically the consistency of the calmness and sense of focus while doing physical activity. 

### The Processing of Self-Compassion Components 

Although mindfulness, self-kindness, self-judgment, common humanity, and self-isolation are components of self-compassion, the outcomes of the interviews showed that the components also formed a series of processing stages, mediated by self-reflection. 

A leisure-based PA participant (*LDF1*) stated, “When doing exercise on my own, like playing basketball or going for a run, that’s a time for me to contemplate, it is a process for me to quietly reflect myself. (How about after reflecting?) I then acknowledge that there may be an issue and try to improve it, or think how to do it better next time”.

It can thus be seen that individuals achieved mindfulness through the process of contemplation during PA and gained self-awareness of their deficiencies. Then, they would reflect and show themselves self-kindness by accepting the deficiency and courageously finding ways to improve, including approaching peers or coaches for their opinions. 

Several participants who engaged in acts of self-judgment also went through the self-compassion processing stages; additionally, they displayed a release of pressure and stress, which can be regarded as resulting in better mental well-being. A leisure-based PA participant who fenced (*LDF6*) reflected as follows: 

I become more persistent or could be called obsessed with my stuff after I have fenced. Fencing is an activity which requires different kinds of postures and these postures must be accurate; therefore, after I have fenced, I tend to be obsessed with those postures that I have done badly and may feel desperate when I keep thinking about why I can’t I do the postures right. (Which means you will also become more persistent while doing other things and will also be obsessed when you do other things wrong?) Yes. (So, how would you overcome this obsession?) I will find my peers or even my coach to talk about it, then I will feel released that, oh, actually everyone will do it wrongly at the beginning, then I will stop being obsessed. 

A school-based PA participant (*WGF5*) also stated: 

I will blame or judge myself after a poor performance in a competition, maybe I will be sad or down for a couple of days after the competition, after that, I will try find my peers to talk about it to see how I can improve in the next competition. I think you definitely would blame yourself when you face difficulties, however, you also need to try finding ways to make a change, but not keep blaming yourself. I think it’s just a process. 

The study’s outcomes indicated that after the process of acknowledging one’s negativities or the particular issue, as well as reflecting on them, individuals who impose self-judgment regarding their failings would blame or criticize themselves for a shorter period of time. The period is not that long for most of the participants. Then, they would engage with common humanity in order to release their negative feelings. However, there was a minority among the participants who self-isolated instead. Additionally, the participants indicated that the experiences and feelings gained from doing PA had changed their way of thinking and attitude towards their daily life issues by osmosis. Despite professional athletes having to pay more attention to their sports performance, participants stated that they held similar attitudes when facing other daily issues, especially when facing academic-related issues. There were, thus no perceivable differences between leisure-based PA participants, school-based PA participants, and professional athletes. Furthermore, when discussing topics related to self-kindness and self-judgment, interviewees were asked whether they were used to thinking positively or negatively about daily issues. Responses showed that participants who typically thought positively and who were optimistic tended to show self-kindness and common humanity, while participants who typically thought negatively and were always being distracted by negative emotions showed self-judgment and isolation, as well as being less mindful. Therefore, personality traits can be regarded as moderating between PA and self-compassion.

## 11. Body Compassion

Body compassion combines the multidimensional concept of body image with self-compassion and indicates an individuals’ level of compassion and acceptance towards their body in terms of appearance, competence, and health.

The interviews outcomes in this study showed participants changed their body perception after indicating their exercise self-efficacy. Participants who showed changes in exercise self-efficacy after physical injuries tended to show changes in body compassion. A school-based PA participant (*LDF5*) stated:

I had suffered from a muscle sprain about one year ago and stopped training for around half a year. I feel like I need to work extra hard to recover and build up the strength of that particular part of the muscle in order to get back to good physical condition for training. Therefore, I keep actively finding ways to solve this problem like consulting the physiotherapist.

Another school-based PA participant (*WGF5*) said, 

I had suffered some small injuries before, not that serious, and I don’t think it had affected my efficacy in doing exercise or recovering. Therefore, I just try to take some rest and won’t consider much about it, everyone will get injuries when doing exercises.

A leisure-based PA participant (*WGF3*) stated, 

I will keep being active despite having some slight injuries in order to improve my body, as I know what kind of exercise I need to do to tackle that injury. For example, I get injured in my back, you know that’s already happened then you may choose some gym exercise that could train and strengthen the back muscles.

A professional athlete participant (SJ2) expressed the following:

There’s a time I competed in a race with my hand injury, I keep mentally telling myself to just go through it even though it is painful. Surprisingly, I even got satisfying results in that race. Therefore, I think it just about your mentality sometimes, but not exactly the physical injuries that affect you. 

According to the statements above, individuals with positive self-efficacy, in spite of injuries, showed positive body compassion by accepting their injuries and acknowledging that injuries resulting from physical activity are common, and by actively finding ways to strengthen the injured body part, or even to break through the mental blocks due to the injuries and regain their reduced exercise efficacy.

However, there were senior form participants who showed negative exercise self-efficacy, as well as negative body compassion, especially defusion. Two professional athletes (*SJ1*, *LDF7*) stated that they had thought of giving up an event or even their sport after a serious injury, while a school-based PA participant (*WGF9*) stated, “Without playing volleyball for more than half a year, my physical fitness and ball sense reduced, and I felt mentally so tired that I thought of just letting it go, just giving up”.

These individuals showed that they tended to want to escape from the fact that they were suffering from an injury, instead of accepting the injury and finding positive ways to treat the injury. Compared to junior form participants who got injured due to physical activity, they showed more kindness and acceptability towards their injuries. They accepted the fact that they were injured, yet they also believed that they would be able to recover and reengage in physical activity in a short period of time.

**Figure 5:**
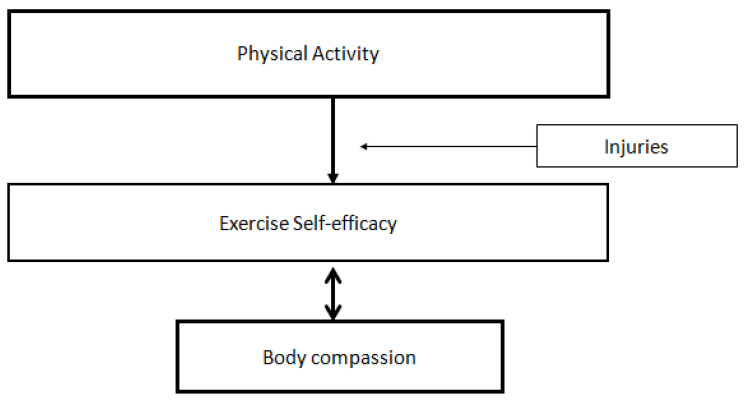
The Relationship between Exercise Self-efficacy and Body Compassion. Note. This figure indicated the path from physical activity to body compassion, which it is in lined with the EXSEM, with the replacement of body compassion. However, injuries is indicated as a possible mediator between physical activity and exercise self-efficacy, in order to reach body compassion. Besides, the relationship between exercise self-efficacy and body compassion could be indicated as inter-related.

A few participants, regardless of age, who did not suffer from injuries due to physical activities gave positive comments relating to body compassion and further demonstrated positive effects towards exercise self-efficacy (Figure 5). For example, a participant who engaged in both leisure-based and school-based PA (*WGF4*) stated, “Doing exercise, both cycling and volleyball, makes my muscles stronger and the shape of my leg muscles more appealing as well. But, as a girl, I don’t think it bothers me, in the opposite, I tend to like this appearance and would like to keep it by keeping on doing exercise”. Another school-based PA participant (*WGF5*) expressed the following:

Since I started to exercise from a young age, I felt that my physical fitness or cardiorespiratory function was better than others, because I have been doing sports for a long period of time. Hence, I felt more confident about my physical body, my exercise skills sometimes, which could help me perform better in a competition. So, I think doing exercises is valuable to my physical body. 

However, the rest of the participants (*LDF1*, *LDF4*, *LDF6*, *WGF1*, *WGF2*, *WGF4*), who did not suffer from any physical injuries, only provided general comments regarding changes in their body, such as being physically stronger and having a better physical appearance, like being slimmer and looking healthier, while showing a neutral perception towards body compassion. These participants expressed that they felt no special thoughts about the value of their physical body and were less likely to relate their physical body with their inner emotions or thoughts.

## 12. Discussion

**Figure 6:**
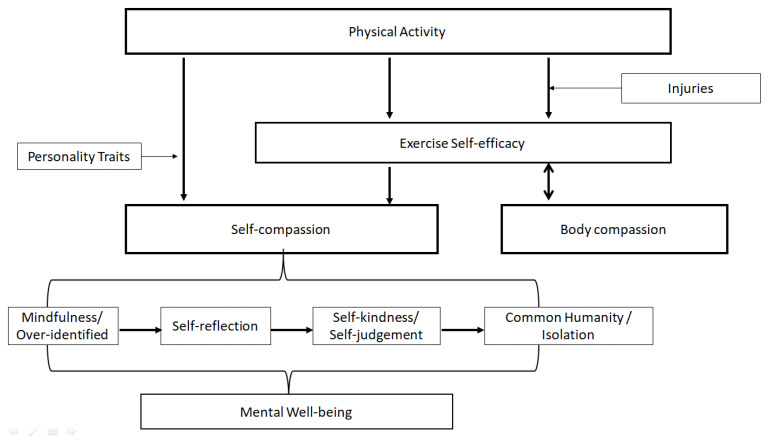
Exercise and Self-Esteem Model revised with self-compassion: An Abductive Qualitative Approach. Note. This figure demonstrates the final model of the interview outcomes. Self-compassion and body compassion have shown as two separate paths, without any inter-relationships. While, the figure also indicates the effect of physical activity and self-compassion in achieving mental well-being.

The study results indicated that the relationship between physical activity and self-compassion could be demonstrated by the EXSEM-SC, with a satisfactory goodness-of-fit index in the SEMs, as well as satisfying model construct consistency. Moreover, the study results showed no significant differences in the level of physical activity, self-compassion, and mental well-being during and after the peak of COVID-19, which, on the one hand, shows the insignificant impact of COVID-19 on physical activity and psychological status among Hong Kong adolescents, and, on the other hand, documented the stability and reliability of the EXSEM-SC and EXSEM-SC with mental well-being model among the Hong Kong adolescents population. The qualitative interview allows the hypothesized model to be linked with the ordinary experiences of the participants, which showed an interactive relationship between the data and the hypothesized EXSEM revised with self-compassion model that has combined with the existing EXSEM model [61,62]. The results demonstrated that the relationship between physical activity and self-compassion was mediated by exercise self-efficacy, which is expected based on the Exercise and Self-Esteem Model revised with self-compassion. Moreover, a new category, personality traits, was determined inductively from the outcomes and seen as a potential moderator in the direct association between physical activity and self-compassion. Self-compassion and the processing stages of self-compassion were shown to achieve further mental well-being as well. Since the body compassion category showed no interrelation between physical activity and self-compassion, a separate conceptual pathway was built between physical activity and body compassion through exercise self-efficacy. Additionally, a new category, injuries, was inductively determined from the outcomes and seen as a potential moderator between physical activity and exercise self-efficacy, which then influenced body compassion. Figure 6 shows the conceptual framework of the Exercise and Self-Esteem Model revised with self-compassion based on the abductive qualitative approach.

### 12.1. The Impact of COVID-19

Surprisingly, the current research showed no significant differences in adolescents’ PA levels during and after the peak of COVID-19. In light of the fact that most of the research studies report significant declines in physical activity levels and healthy lifestyle behaviors [17], it was anticipated that external factors would play a role in helping Hong Kong adolescents maintain their PA levels. Research demonstrated that parents’ education and parental support on the importance of maintaining healthy living during public health challenges were positively correlated with adolescents’ PA level and maintained a sufficient PA level during COVID-19 [63]. In addition, environmental and psychosocial factors are considered to be critical in supporting adolescents’ intrinsic motivation to engage in physical activity and their self-determination to pursue positive well-being [64]; therefore, based on the self-determination theory, adolescents should have been able to engage in physical activity autonomously under the support of schools and parents despite social distancing measures [65]. Based on empirical research, adolescents are likely to have scheduled their daily schedule, which includes engaging in various hobbies and physical activities, is well-organized, and reflects their adaptive coping abilities, contributing to subjective well-being [66]. Hence, it supports that people with a high level of social cognition, including self-efficacy and self-regulation, showed a higher level of PA during the COVID-19 pandemic as well.

As a result of these empirical findings, it can also be explained that Hong Kong adolescents’ exercise confidence has increased as foreseeable obstacles to exercise engagement or other exercise barriers have been reduced as a result of COVID-19, and they are able and feel more confident to engage in physical activity with more time and capacity. Moreover, adolescents at the transformation stage of maturation and risk of identity crisis [67] are expected to have heightened attention towards health literacy and body image, thus resulting in engaging in preventive behaviour, such as physical activity, as well as demonstrating a higher level of exercise self-efficacy and body compassion. During COVID-19, despite there being research that indicated negative body image in women due to struggling with regular eating habits [68], another study indicated that students showed a decrease in unhealthy food intake and irregular eating habits [69]. In this way, Hong Kong adolescents are more likely to show kindness and compassion to their bodies and to realize that others may also be experiencing similar struggles during the lockdown or social isolation measures associated with COVID-19.

Apart from the unchanged PA level, Hong Kong adolescents’ self-compassion was less likely to be affected by the COVID-19 pandemic. As mentioned in the literature review, self-compassion could act as a protective role and a positive coping strategy during COVID-19, resulting in better life satisfaction [13]. Moreover, empirical research studies demonstrated that adolescents with a higher level of self-compassion showed a direct association with psychological well-being [70], in which one would engage in reducing stress and fears brought by COVID-19 and show gratitude towards their concurrent life status [70]. However, there was limited research stating the impact of COVID-19 on self-compassion, except those of intervention studies. Self-compassion was less likely to be affected by COVID-19 because it is conceptualized as a psychological trait. It has always been found that self-compassion acts as an emotional protective factor for mental health problems, such as depression and anxiety, and that self-compassion measures are consistent across populations across time [71]. Therefore, self-compassion was conceptualized as a trait model similar to that of personality traits [72], which were commonly examined together in interpersonal and intrapersonal problems [71,73,74,75]. However, self-compassion is a modifiable trait [76]; it can only be cultivated or improved through intervention or practice, but is less likely to be affected by COVID-19 significantly.

### 12.2. The Newly Derived Variables from the Qualitative Interview

#### 12.2.1. Personality Traits Moderating Physical Activity and Self-Compassion

The research outcomes showed that individuals with a more positive and optimistic mindset tended to demonstrate positive self-compassion; vice versa, individuals who were perfectionists and who tended to be obsessed with their failings demonstrated negative self-compassion. Hence, it could be interpreted that individual differences involving personality traits would affect the relationship between physical activity and self-compassion. The literature contains limited research regarding physical activity and self-compassion, never mind involving personality traits. Yet there was sufficient research stating the effect of personality on self-compassion. Research has shown that perfectionism reduced levels of self-compassion and, thus, increased the risk of depression [77], and this moderating relationship was shown to be more significant among adolescents [78]. People with personality disorders, including narcissistic and borderline personality disorder, showed a negative association with self-compassion and a positive association with self-criticism. Based on the Big Five personality traits, self-compassion was once regarded as being similar to neuroticism, hence neuroticism was tested and demonstrated a significant correlation with negative self-compassion [79]. Furthermore, other personality traits, including extraversion, agreeableness, conscientiousness, and openness, as well as their latent factors, demonstrated a significant positive correlation with positive self-compassion [79,80]. According to the above literature, personality traits can be regarded as a factor influencing individuals’ levels of self-compassion. Despite physical activity having been shown to be a self-care method to improve self-compassion, the degree of improvement may be influenced by one’s personality. Therefore, other than including personality traits as a testing component in the model, the potential effect of personality traits should be considered as an influencing factor or a covariance in future physical activity intervention research in order to identify the potential differences among personality traits regarding the effect of physical activity on self-compassion.

#### 12.2.2. The Stages of Processing within Self-Compassion through Self-Reflection 

The process cycle of the self-compassion facets has been discussed by Neff [27], showing that individuals with self-compassion could first mindfully acknowledge and become aware of their own sufferings, then alleviate them with kindness, while gentle comfort and soothing helps individual to accept their failings by relating themselves to others and the outside world. However, in Neff’s [27] article, she only briefly mentioned the process of self-reflection together with self-kindness and self-judgement, but did not consider self-reflection as a separate stage within self-compassion. Moreover, a study of Chinese adults indicated that self-kindness and mindfulness could moderate the relationship between self-criticism and depression [81]. The current study, however, indicates that self-reflection is a perceivable stage within the three facets of self-compassion, in particular between mindfulness/over-identified and self-kindness/self-judgment. Participants demonstrated that they would process self-reflection first, regardless of whether it was gentle or cruel, about their failings and deficiencies, then they expressed their way of perceiving the deficiencies, as well as the reflections. Research has shown that individuals with increased mindfulness were associated with better self-awareness and self-reflection, thus leading to an increase of self-compassion [82]. This is because being mindful helps individuals to discover their boundaries easily and equips them with better self-knowledge, thus leading to better performance in self-acceptance and showing gentleness towards oneself [62,83]. Additionally, self-reflection was also regarded as a tool to emphasize oneself, where individuals who have performed self-reflection demonstrated positive emotions and enhanced the positive soothing effect, thus representing the achievement of self-kindness [84]. 

#### 12.2.3. The Association between Body Compassion, Self-Compassion, and Psychological Status among Hong Kong Secondary School Students

Based on the significant association between body compassion and self-compassion reported in previous studies [24], it was expected that Hong Kong secondary school students would express a perceivable relationship between body compassion and self-compassion. Although a few participants mentioned this aspect, they showed a common feature in which was they all treated physical injuries and the effect on their physical activity engagement as the most important issue is their life, which showed a recognizable relationship within their conversation. Research studies have also supported that people with physical injuries would be paying more attention towards their body image and self due to bodily changes, and resulted in either positive or negative self-concept and body image [85]. Furthermore, a recent study indicated that illness and injuries had been shown to influence and cause variation in the relationship between body and physical activity. In particular, people who engaged in regular physical activities would tend to recall criticism or to reflect on their bodily change, as well as would intend to manage injury setbacks in sports [86]. Therefore, it supported the role of injuries in affecting the relationship between physical activity and body compassion.

Moreover, despite Hong Kong secondary school students being less likely to relate their physical body with their inner self, they experienced a release of distress and pressure after having engaged in physical activity, as well as having gone through the process of self-compassion. A systematic review and meta-analysis [87] revealed that physical activity intention and physical activity engagement were positively correlated with self-compassion. Furthermore, research studies that targeted Chinese college students indicated that individuals with a high level of self-compassion were negatively correlated with anxiety and depression and positively correlated with subjective well-being [88,89,90]. As already mentioned, Hong Kong adolescents displayed the processing cycle of achieving self-compassion, in which individuals with self-judgement would show common humanity for seeking references from the outside world. It is worth noting that one research study has reported that common humanity was shown as the moderator in the relationship between self-criticism and depression [81], which further supports the processing cycle of self-compassion revealed by the current study and, at the same time, further extending the Exercise and Self-Esteem Model revised with self-compassion by indicating the association with mental well-being.

### 12.3. Limitations

Regardless of the quantitative results showing consistent results over time, the qualitative interview revealed that most participants had a neutral attitude toward body compassion, acknowledging that physical activity engagement would contribute to improved exercise self-efficacy and body image, but are less likely to care about their body image or its effects on mental health. Although sex has been shown to play a significant role in body-related issues, including body satisfaction, female participants in this study showed no differences compared with male participants. As a result, this qualitative study could barely determine the relationship between body compassion and self-compassion. In fact, within the scarce literature regarding Chinese adolescence body image, research studies have demonstrated that self-compassion played a predictive effect on body dissatisfaction, which helped reduce body dissatisfaction and negative mental well-being [91]. Moreover, one study showed that individuals with a high level of self-compassion showed no association between body shame and depression [92]. According to the research outcomes, the majority of the participants tended to be optimistic and with positive self-compassion, which might explain the unexpected relationship between body compassion and self-compassion; yet, further investigation is needed.

### 12.4. Strengths and Applications 

This current research indicated that Hong Kong adolescents’ physical activity, self-compassion, and mental well-being were stable during the COVID-19 pandemic. Moreover, despite such tremendous daily life routine changes, the EXSEM-SC and EXSEM-SC with mental well-being model were still able to show notable statistical stability. This reveals the reliability of the models as well as the solidity of the relationship between physical activity and self-compassion. Therefore, owing to the significant mental health problems and high suicidal rate among Hong Kong adolescents regardless of the COVID-19 pandemic, it is expected that the physical activity intervention, which is based on the EXSEM-SC model, could also aim at easing Hong Kong adolescent’s mental health issues. In addition, according to the Organisation for Economic Co-operation and Development (OECD), promoting well-being is being one of the key competencies of the global educational trend, while Hong Kong secondary education has also stated that mental well-being is being one of the components in the healthy lifestyle curriculum; therefore, in terms of generating a long-term impact, the physical activity and self-compassionate intervention should be promoted among schools through advocating as extra-curriculum and further being involved in regular physical education lessons. Furthermore, the stand-alone physical activity intervention program could also involve self-compassion psycho-educational components to further enhance its effect on improving self-compassion and mental well-being, then to disseminate through developing a manual for enhancing the health education materials in the future. Last, but not least, with the stability of the EXSEM-SC model among adolescents, the model can be applied to other populations, including older adults, young children, and athletes. Hence, it could be further explored and investigated the association between physical activity and self-compassion among other populations with mental health issues.

## 13. Conclusions

Using the Exercise and Self-Esteem Model revised with self-compassion, the current study has revealed the relationship between physical activity and self-compassion among Hong Kong secondary school students, as well as the steps toward mental wellbeing. Exercise and Self-Esteem Model is a well-known and well-established model, along with the self-concept model, that illustrates how exercise is linked to self-esteem. Self-compassion may be in line with the self-concept model, according to this study. In this research, physical activity is shown to contribute to self-compassion, both directly and indirectly, thus encouraging the use of physical activity as a tool toward self-compassion.

## Figures and Tables

**Table 1:** Demographic Information of the in-depth interview.

Participant’s Code ID	Age	Gender	Form of Physical Activity	Type of Physical Activity
CKS1	14	Male	School-based	Table Tennis
CKS2	14	Male	Leisure-based	Swimming
CSK3	13	Male	Leisure-based	Swimming
CSK4	13	Male	Leisure-based & School-based	Running & Basketball
CSK5	12	Male	School-based	Basketball
CSK6	14	Male	School-based	Taekwondo
WGF1	16	Female	Leisure-based	Volleyball
WGF2	16	Male	Leisure-based	Basketball, Football & Running
WGF3	16	Male	Leisure-based	Running & Cycling
WGF4	16	Female	Leisure-based & School-based	Yoga & Volleyball
WGF5	16	Male	Leisure-based & School-based	Softball & Gym
WGF6	14	Male	School-based	Football
WGF7	14	Female	Leisure-based	Cycling
WGF8	14	Female	Leisure-based & School-based	Table Tennis & Volleyball
WGF9	14	Female	School-based	Table Tennis & Volleyball
WGF10	14	Female	Leisure-based& School-based	Table Tennis & Volleyball
LDF1	15	Female	Leisure-based	Basketball & Running
LDF2	15	Female	Leisure-based	Basketball & Cycling
LDF3	15	Female	Leisure-based	Basketball
LDF4	15	Male	School-based	Taekwondo
LDF5	15	Male	School-based	Fencing
LDF6	15	Male	Leisure-based	Fencing
LDF7	18	Female	Professional Athletes	Swimming
SJ1	18	Male	Professional Athletes	Swimming
SJ2	15	Male	Professional Athletes	Swimming

**Table 2:** Paired T-test.

		Mean Differences	Std. Deviation	t	df	Sig.
Pair 1	PA & R_PA	−0.02	0.92	−0.67	568	0.50
Pair 2	ESE & R_ESE	−0.3	2.57	−3.14	568	0.002
Pair 3	SC & R_SC	0.0	0.70	1.63	568	0.10
Pair 4	BC & R_BC	−0.10	0.69	−3.33	568	0.001
Pair 5	WB & R_WB	0.036	1.06	0.80	568	0.42

Note: PA = Time 1 physical activity; R_PA: Time 2 physical activity; ESE = Time 1 exercise self-efficacy; R_ESE = Time 2 exercise self-efficacy; SC = Time 1 self-compassion; R_SC = Time 2 self-compassion; BC = Time 1 body compassion; R_BC = Time 2 body compassion; WB = Time 1 mental well-being; R_WB = Time 2 mental well-being.

**Table 3:** Correlation Matrix Among All Variables Between Test and Re-Test.

	PA	R_PA	ESE	R_ESE	SC	R_SC	BC	R_BC	WB	Mean (*SD*)
PA	-									2.20 (0.78)
R_PA	0.30 **	-								2.18 (0.76)
ESE	0.57 **	0.15 **	-							4.62 (2.15)
R_ESE	0.17 **	0.54 **	0.24 **	-						4.96 (2.00)
SC	0.10 *	0.04	0.24 **	0.11 **	-					3.10 (0.50)
R_SC	0.06	0.25 **	0.11 *	0.38 **	0.14 **	-				3.05 (0.57)
BC	0.08 *	0.02	0.24 **	0.10 *	0.618 **	0.113 **	-			3.01 (0.47)
R_BC	0.01	0.15 **	0.004	0.20 **	0.08	0.57 **	0.09 *	-		3.11 (0.55)
WB	0.21 **	0.13**	0.23 **	0.15 **	0.51 **	0.15 **	0.48 **	0.09 *	-	3.30 (0.85)
R_WB	0.04	0.31 **	0.04	0.39 **	0.11 *	0.49 **	0.07	0.48 **	0.19 **	3.26 (0.82)

Note: * *p* < 0.05, ** *p* < 0.01. PA = Time 1 physical activity; R_PA: Time 2 physical activity; ESE = Time 1 exercise self-efficacy; R_ESE = Time 2 exercise self-efficacy; SC = Time 1 self-compassion; R_SC = Time 2 self-compassion; BC = Time 1 body compassion; R_BC = Time 2 body compassion; WB = Time 1 mental well-being; R_WB = Time 2 mental well-being.

## Data Availability

Data will be available upon reasonable request.
